# Supplementary material for: Inflammation induced by incomplete radiofrequency ablation accelerates tumor progression and hinders PD-1 immunotherapy
Source: Nat Commun. 2019 Nov 28;10:5421. doi: 10.1038/s41467-019-13204-3 (PMC6883042; doi:10.1038/s41467-019-13204-3)
Supplement: Supplementary file 1 — Supplementary Information [file 41467_2019_13204_MOESM1_ESM.pdf]

Supplementary Information

Inflammation induced by incomplete radiofrequency ablation accelerates tumor progression and hinders PD-1 immunotherapy

Shi et al.

Supplementary Table 1. Baseline of characteristics

Characteristics	Group A (n=43)	Group B (n=43)	*P* value
Age (year)			0.675
Median,	62	61	
Range	32-78	39-78	
Gender			1.000
Male	26	26	
Female	17	17	
Performance status			0.661
0	24	26	
1	19	17	
Site of primary cancer			0.784
Right colon	15	18	
Left/sigmon colon	12	10	
Rectum	16	15	
Time to liver metastases			0.805
Synchronous	12	10	
Metachronous	31	33	
Time size			0.067
≤3 cm	19	10	
>3 cm	24	33	
Number of lesions			0.910
1	21	19	
2–3	13	14	
3–5	9	10	
Extrahepatic metastases			0.757
With	5	7	
Without	38	36	
Baseline of CEA			0.666
≤200 ng/mL	22	19	
>200 ng/mL	21	24	

Abbreviations: CEA, carcinoembryonic antigen

Supplementary Table 2. Adverse tumor factors affecting RFA operation in patients with local residual tumor

Factors	n
Tumor size>3cm	14
Contacting with portal vein	14
Contacting with hepatic hilum	10
Adjacent to the gastrointestinal tract	6
Adjacent to the gallbladder	2

Three patient had tumor with 3 adverse factors.

Supplementary Table 3. Multivariate analysis of prognostic factors for time to new metastasis and survival after RFA

Factors	TTNM			OS		
	B	SE	*P* value	B	SE	*P* value
Site of primary cancer	0.071	0.135	0.600	0.025	0.145	0.864
CEA	0.171	0.130	0.187	0.143	0.133	0.280
Time to liver metastases	0.473	0.244	0.075	0.346	0.287	0.228
Number of lesions	0.191	0.155	0.218	0.418	0.161	0.010
Tumor size	0.612	0.244	0.012	0.764	0.252	0.002
Incomplete ablation	1.118	0.262	<0.001	1.205	0.273	<0.001

Source data are provided as a Source Data file.

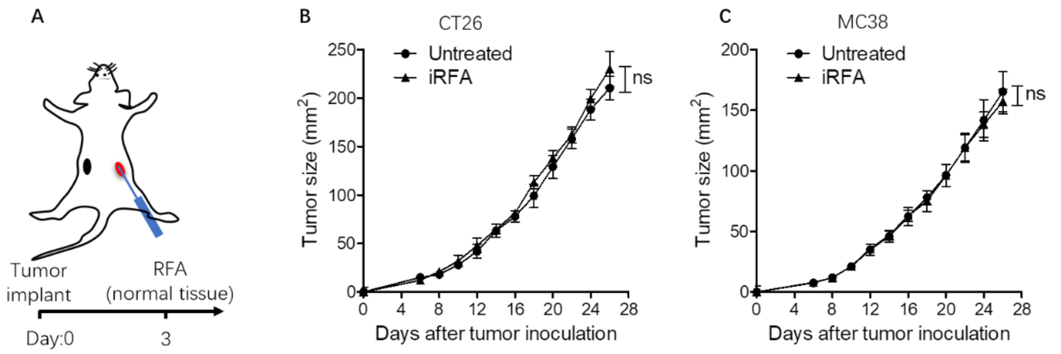

Supplementary Figure 1. Thermal injury in normal tissue caused by RFA does not lead to tumor progression. **A.** Schematic of the study. CT26 cells were injected *i.d.* into male Balb/c on the left flank. RFA was performed to cause thermal injury in normal tissue at the right flank (n=5). **B.** Growth curve of the tumor at the left flank (n=5). Each error bar represents means±SEM, One-sided ANOVA test, ns present not significant. Source data are provided as a Source Data file.

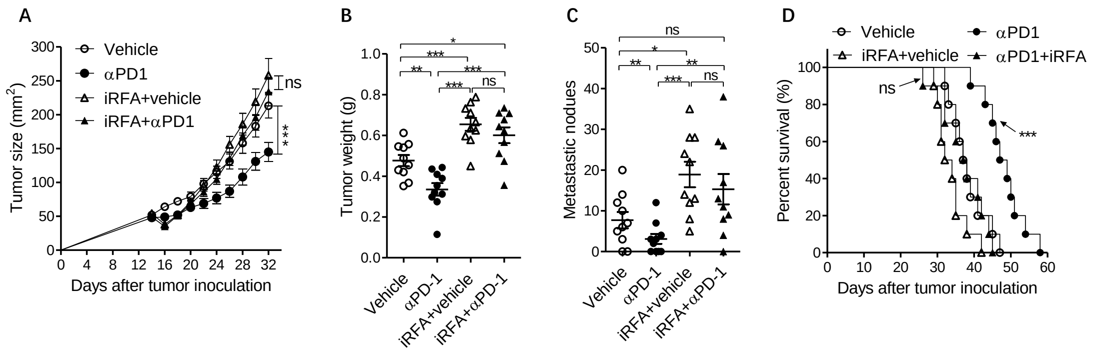

Supplementary Figure 2. iRFA promotes tumor progression and hinders the effect of anti-PD-1 therapy in Hepa1-6 hepatic cancer mouse model. Hepa1-26 model treated with iRFA and anti-PD-1 mAb as descripted in Fig 2F. **A.** Growth curve of the residual tumor (n=10). **B.** Weight of the residual tumor examined on day 14 after iRFA by dissection the mice (n=10). **C.** Number of the metastasis examined on day 14 after iRFA by dissection the mice (n=10). **D.** Kaplan-Meier survival curves are shown and the log-rank test was performed (ns: compared to iRFA+vehicle, ***: compared to the other three groups, n=10). Data represent cumulative results from 1 of 2 independent. The data are represented as mean±SEM. Statistical differences between pairs of groups were determined by a two-tailed Student's t-test (values represent means±SEM, ns present not significant, *P <0.05, **P <0.01, ***P <0.001). Source data are provided as a Source Data file.

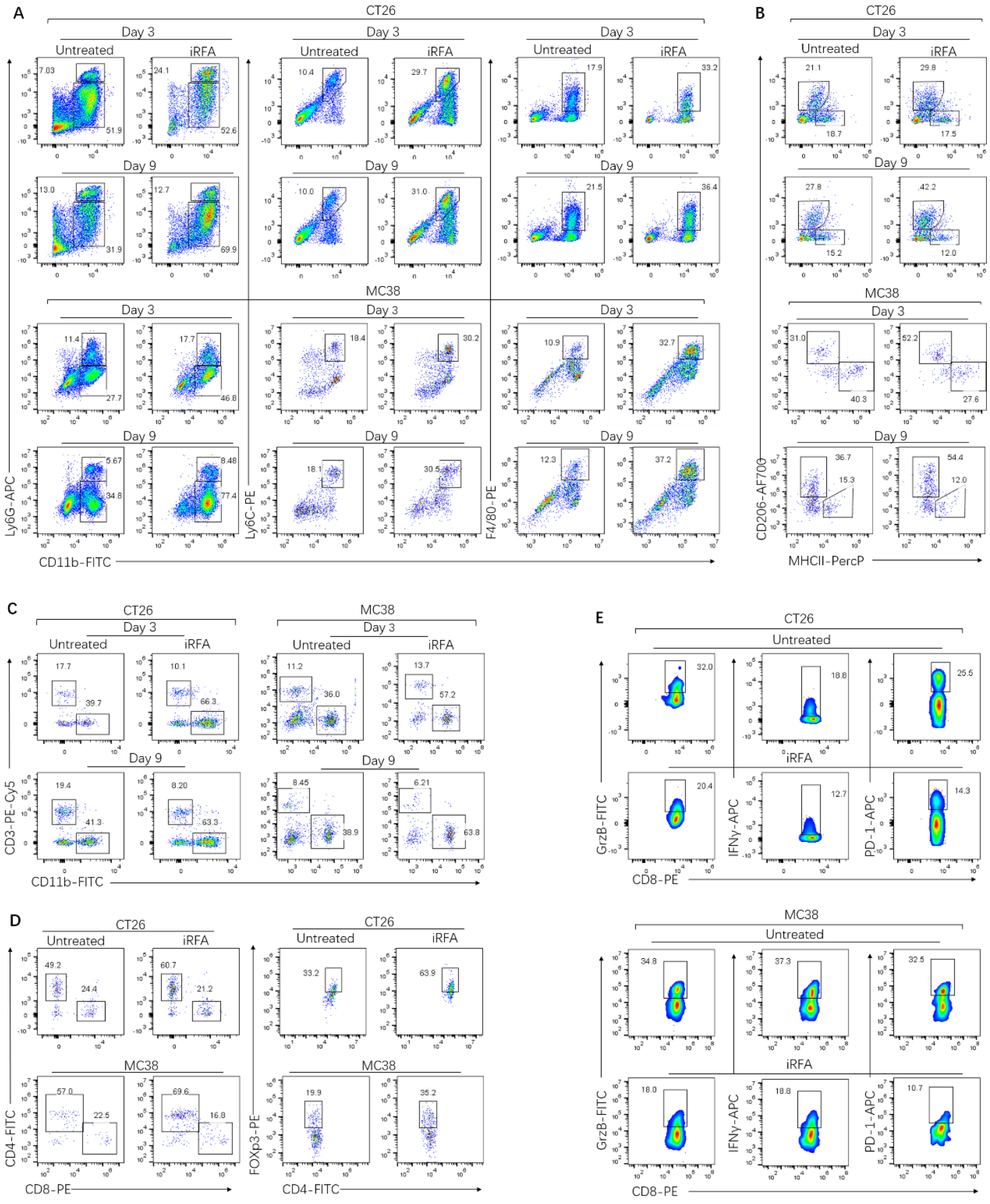

Supplementary Figure 3. Flow cytometric analysis of myeloid cells infiltration and T response in the residual tumors. iRFA was administrated in CT26 and MC38-bearing mice as described in Fig. 2A. On days 3 and 9 after iRFA treatment, the residual tumors were resected and either digested to generate single cell suspension or to isolate RNA or embedded in paraffin. **A.** Representative flow cytometric analysis of $CD11b^+$, $CD11b^+Ly6G^+$, $CD11b^+Ly6C^+$, and $CD11b^+F4/80^+$ cells (gate on $CD45^+$ liver cells) in the untreated and iRFA-treated CT26 and MC38 tumors (n=5). **B.** Representative

flow cytometric analysis of CD11b$^+$F4/80$^+$ (TAM) population, and expression of CD206 and MHCII in CD11b$^+$F4/80$^+$ cell populations in the untreated and iRFA-treated CT26 and MC38 tumors (n=5). **C.** Representative flow cytometric analysis of CD11b$^+$, CD3$^+$ cells in the untreated and iRFA-treated CT26 and MC38 tumors on day 3 and day 9 (n=5). **D.** Representative flow cytometric analysis of CD8$^+$, CD4$^+$ and FoxP3$^+$ cells in the untreated and iRFA-treated CT26 and MC38 tumors on day 3 and day 9 (n=5). **E.** Representative flow cytometric analysis of granzyme B, IFN-γ and PD-1 expression in CD8$^+$ cells in the untreated and iRFA-treated CT26 and MC38 tumors on day 3 and day 9 (n=5).

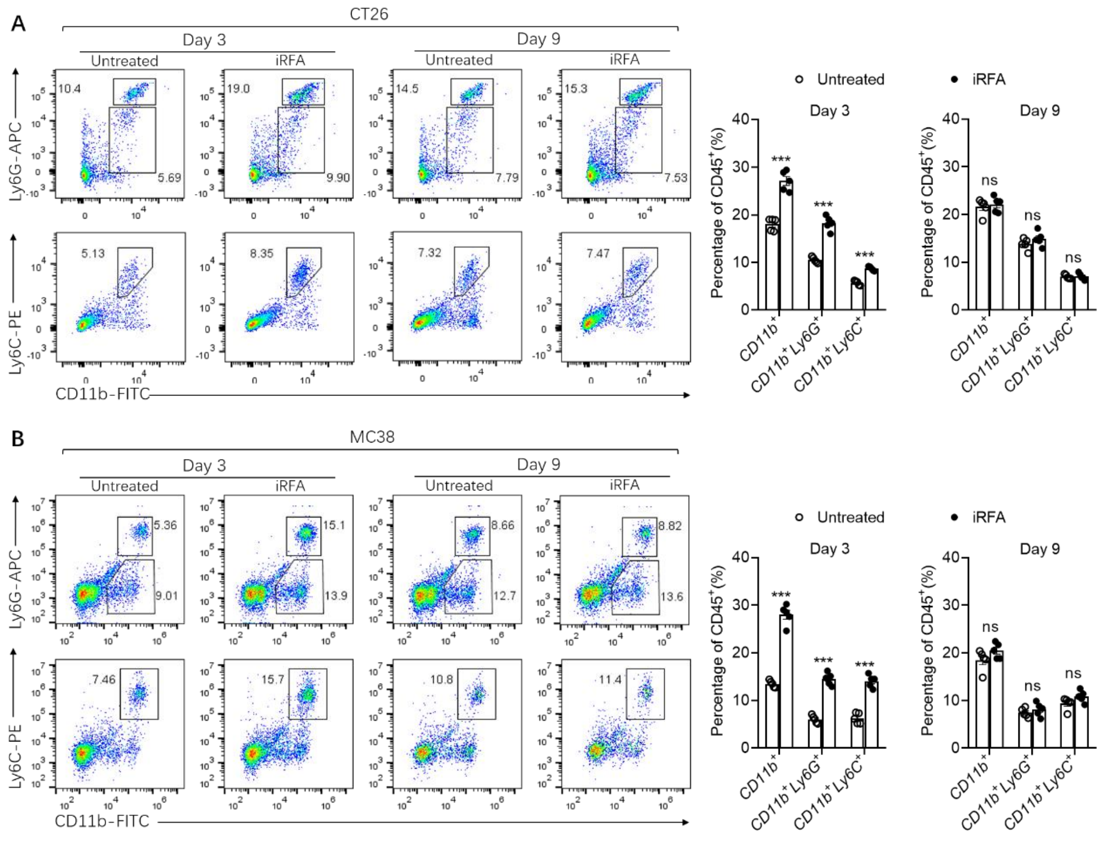

Supplementary Figure 4. iRFA induces a transient increase of neutrophils and monocytes in peripheral blood. iRFA was administrated in CT26 and MC38-bearing mice as described in Fig. 2A. **A.** Flow cytometric analysis of $CD11b^+$, $CD11b^+Ly6G^+$, and $CD11b^+Ly6C^+$ in peripheral blood on days 3 and 9 (n=5). **B.** Percentages of $CD11b^+$, $CD11b^+Ly6G^+$, and $CD11b^+Ly6C^+$ on days 3 and 9. Data represent results from 1/2 independent experiments (n=5). The data are represented as mean ± SEM. Statistical differences between pairs of groups were determined by a two-tailed Student’s t-test (values represent mean ± SEM, ****P*<0.001). Source data are provided as a Source Data file.

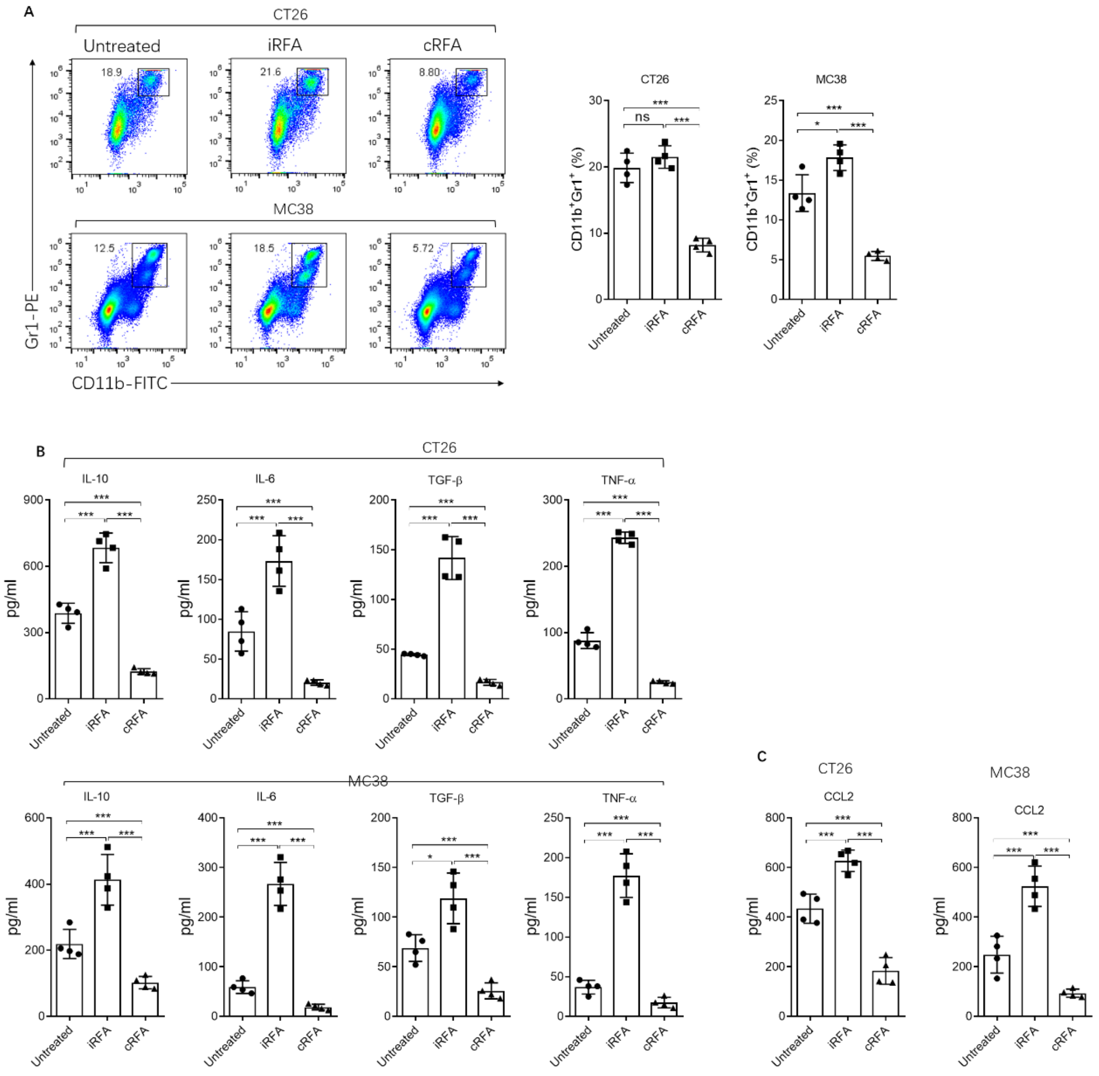

Supplementary Figure 5. The changes of peripheral MDSCs and immunosuppressive cytokines and chemokine after cRFA and iRFA. CT26 and MC38-bearing mice as described in Fig. 1A. cRFA or iRFA was performed when the longest dimension reach about 0.8 cm. The venous blood was obtained from the orbit of mice 9 day after treatment. **A.** Flow cytometric analysis and quantification of $CD11b^+Gr1^+$ cells (n=4). **B.** Measurements of cytokines IL-10, IL-6, TGFβ and TNFα using ELISA kit (n=4). **C.** Measurement of CLL2 using ELISA kit. Data represent results from 1/2 independent experiments (n=4). The data are represented as mean±SEM. Statistical differences between pairs of groups were determined by a two-tailed Student's t-test (values represent mean±SEM, ns presents not significant, *P <0.05, ***P <0.001). Source data are provided as a Source Data file.

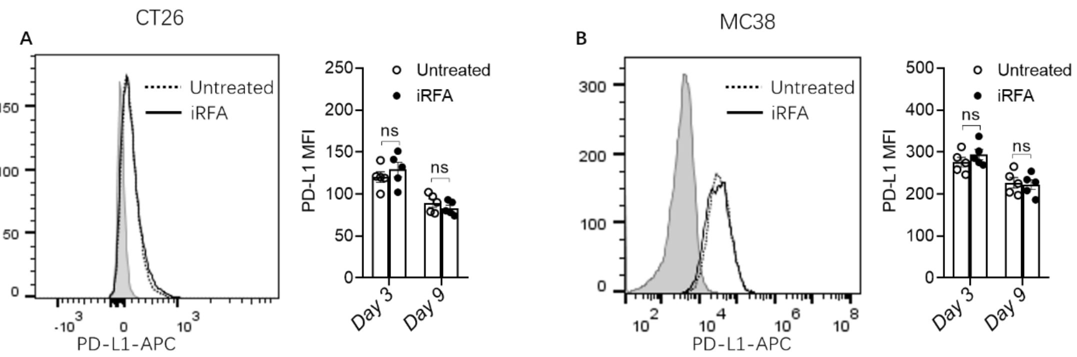

Supplementary Figure 6. PD-L1 expression in macrophages infiltrating into the residual tumor after iRFA. iRFA was administrated in CT26 and MC38-bearing mice as described in Fig. 2A. PD-L1 expression on CD11b^{+}F4/80^{+} cells was analyzed by FCM on day 9 after treatment. Representative histogram and median fluorescence intensity (MFI) were showed. **A.** CT26 model (n=5). **B.** MC38 model. Data represent results from 1/2 independent experiments (n=5). The data are represented as mean ± SEM. Statistical differences between pairs of groups were determined by a two-tailed Student's t-test (values represent mean ± SEM). Source data are provided as a Source Data file.

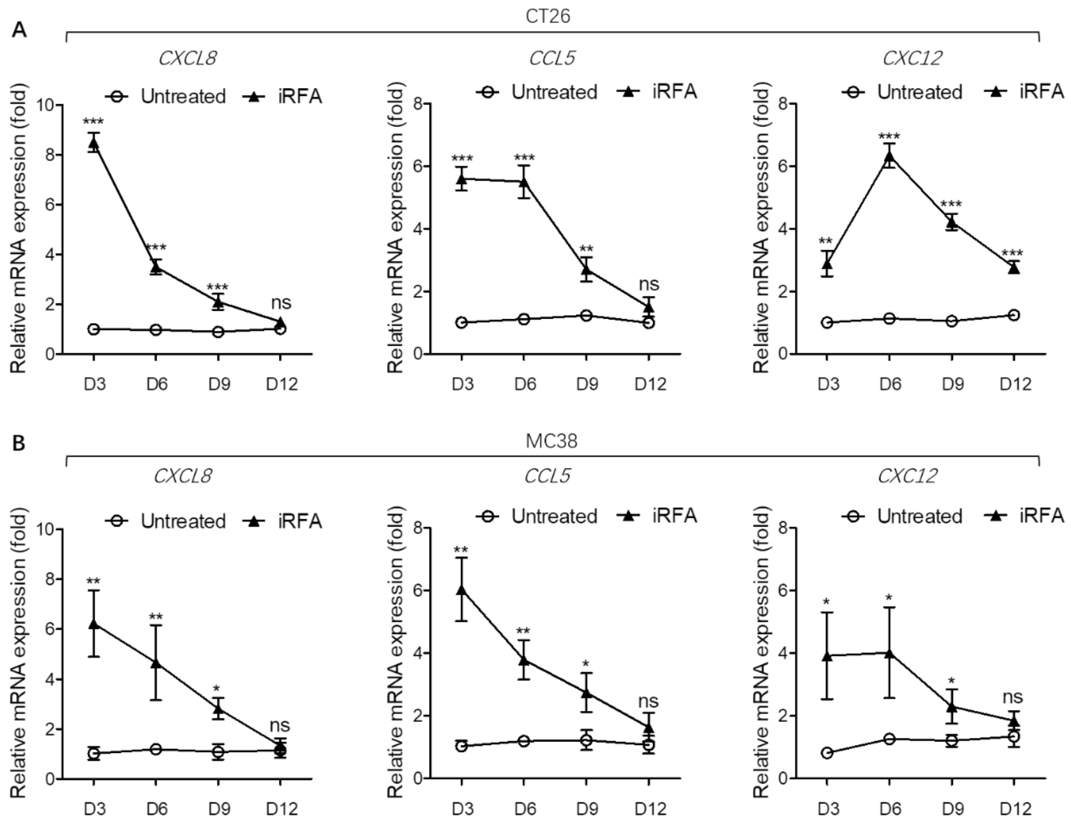

Supplementary Figure 7. Altered level of *CXCL8*, *CCL5*, and *CXCL12* mRNA in the residual tumor. iRFA was administrated in CT26 and MC38-bearing mice as described in Fig. 2A. On days 3, 6, 9, and12 after iRFA treatment, the residual tumors were resected, and total RNA was extracted. *CXCL8*, *CCL5*, and *CXCL12* mRNA expressions were quantified by real-time PCR. Relative mRNA expression was expressed as fold-change. **A.** Data from CT26 model (n=3). **B.** Data from MC38 model. The data are represented as mean±SEM (n=3). Statistical differences between pairs of groups were determined by a two-tailed Student's t-test (values represent mean±SEM, ns presents not significant, **P* <0.05, ***P* <0.01, ****P*<0.001). Source data are provided as a Source Data file.

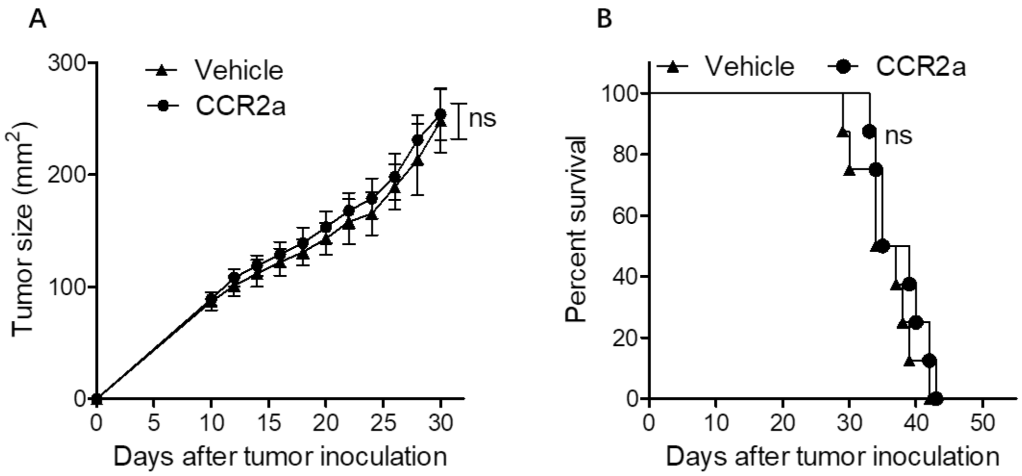

Supplementary Figure 8. Treatment with CCR2 antagonist alone has little effect on the inhibition of the growth of intact CT26 tumor. CT26 cells were injected *i.d.* into male Balb/c on the right flank. When the tumors reach the diameter of about 0.5 cm, CCR2 antagonist (CCR2a) was given subcutaneously at a dose of 5 mg/kg twice per day for 9 days. **A.** Growth curve of the implanted tumors (one-sided ANOVA test, *P*=0.162, n=8). **B** Kaplan–Meier survival curves are shown, and the log-rank test was performed (*P*=0.293, n=8)**.** Source data are provided as a Source Data file.

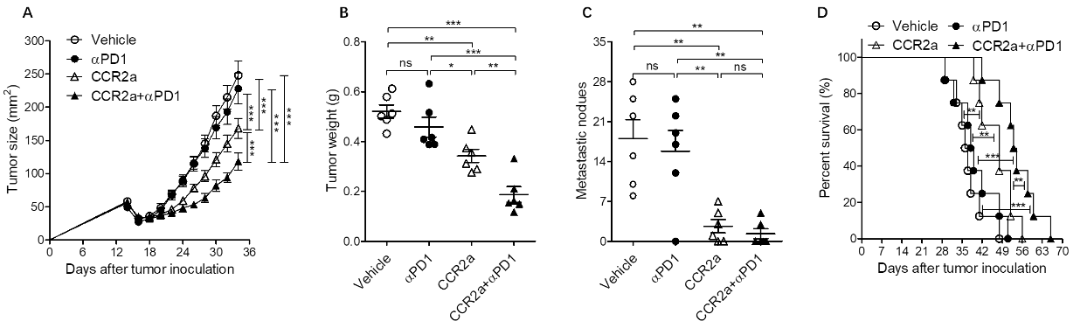

Supplementary Figure 9. Treatment with CCR2 antagonist inhibits progression of residual tumor and overcomes resistance to anti-PD-1 therapy in Hepa1-6 mouse model. The Hepa1-6 tumor-bearing mice models were established and treated with iRFA, CCR2 antagonist (CCR2a) and anti-PD-1 as described in Fig 8A. **A** Growth curve of the residual tumor (one-sided ANOVA test, ****P*<0.001, n=8). **B** Weight of the residual tumor examined on day 14 after iRFA by dissection the mice (n=6). **C** Number of the metastasis examined on day 14 after iRFA by dissection the mice (n=6). **D** Kaplan-Meier survival curves are shown and the log-rank test was performed (n=8). Data represent results from 1 of 2 independent experiments. The data are represented as mean±SEM. Statistical differences between pairs of groups were determined by a two-tailed Student's t-test (ns present not significant, **P*<0.05, ***P*<0.01, ****P*<0.001). Source data are provided as a Source Data file.
