# Supplementary material for: Inflammation induced by incomplete radiofrequency ablation accelerates tumor progression and hinders PD-1 immunotherapy
Source: Nat Commun. 2019 Nov 28;10:5421. doi: 10.1038/s41467-019-13204-3 (PMC6883042; doi:10.1038/s41467-019-13204-3)
Supplement: Supplementary file 3 — Reporting Summary [file 41467_2019_13204_MOESM3_ESM.pdf]

nature research

Corresponding author(s): Weihua Liao

Last updated by author(s): Sep 28, 2019

Reporting Summary

Nature Research wishes to improve the reproducibility of the work that we publish. This form provides structure for consistency and transparency in reporting. For further information on Nature Research policies, seeAuthors & Referees and theEditorial Policy Checklist .

Statistics

For all statistical analyses, confirm that the following items are present in the figure legend, table legend, main text, or Methods section.

n/a	Confirmed	
☐	☒	The exact sample size (*n*) for each experimental group/condition, given as a discrete number and unit of measurement
☐	☒	A statement on whether measurements were taken from distinct samples or whether the same sample was measured repeatedly
☐	☒	The statistical test(s) used AND whether they are one- or two-sided<br>*Only common tests should be described solely by name; describe more complex techniques in the Methods section.*
☐	☒	A description of all covariates tested
☐	☒	A description of any assumptions or corrections, such as tests of normality and adjustment for multiple comparisons
☐	☒	A full description of the statistical parameters including central tendency (e.g. means) or other basic estimates (e.g. regression coefficient) AND variation (e.g. standard deviation) or associated estimates of uncertainty (e.g. confidence intervals)
☐	☒	For null hypothesis testing, the test statistic (e.g. *F*, *t*, *r*) with confidence intervals, effect sizes, degrees of freedom and *P* value noted<br>*Give P values as exact values whenever suitable.*
☒	☐	For Bayesian analysis, information on the choice of priors and Markov chain Monte Carlo settings
☒	☐	For hierarchical and complex designs, identification of the appropriate level for tests and full reporting of outcomes
☒	☐	Estimates of effect sizes (e.g. Cohen's *d*, Pearson's *r*), indicating how they were calculated

Our web collection on statistics for biologists contains articles on many of the points above.

Software and code

Policy information about availability of computer code

Data collection	No software were used.
Data analysis	Data were analyzed using SPSS software (Version 13.0) and GraphPad Prism (version 8.0)

For manuscripts utilizing custom algorithms or software that are central to the research but not yet described in published literature, software must be made available to editors/reviewers. We strongly encourage code deposition in a community repository (e.g. GitHub). See the Nature Research guidelines for submitting code & software for further information.

Data

Policy information about availability of data

All manuscripts must include a data availability statement. This statement should provide the following information, where applicable:

- Accession codes, unique identifiers, or web links for publicly available datasets
- A list of figures that have associated raw data
- A description of any restrictions on data availability

A list of figures that have associated raw data.

Field-specific reporting

Please select the one below that is the best fit for your research. If you are not sure, read the appropriate sections before making your selection.

☒ Life sciences ☐ Behavioural & social sciences ☐ Ecological, evolutionary & environmental sciences

For a reference copy of the document with all sections, see nature.com/documents/nr-reporting-summary-flat.pdf

Life sciences study design

All studies must disclose on these points even when the disclosure is negative.

Sample size	Sample sizes in all studies were determined by power analysis assuming 2-sided significance as 5% at 80% power level.
Data exclusions	No data were excluded for analysis.
Replication	All experimental findings were verified in 2 independent experiments.
Randomization	The experimental mice were grouped by random number.
Blinding	The investigators were blinded to group allocation during data collection and analysis.

Reporting for specific materials, systems and methods

We require information from authors about some types of materials, experimental systems and methods used in many studies. Here, indicate whether each material, system or method listed is relevant to your study. If you are not sure if a list item applies to your research, read the appropriate section before selecting a response.

Materials & experimental systems

n/a	Involved in the study	
☐	☒	Antibodies
☐	☒	Eukaryotic cell lines
☒	☐	Palaeontology
☐	☒	Animals and other organisms
☒	☐	Human research participants
☒	☐	Clinical data

Methods

n/a	Involved in the study	
☒	☐	ChIP-seq
☐	☒	Flow cytometry
☒	☐	MRI-based neuroimaging

Antibodies

Antibodies used	Mouse antibodies to CD45 (clone: 30-F11, eBioscience), CD3 (clone: 145-2c11, eBioscience), CD4 (clone: GK1.5, eBioscience), CD8 (clone: 53-6.7, eBioscience), granzyme B (clone: NGZB, eBioscience), IFN-γ (clone: XMG1.2, eBioscience), FOXP3 (clone: FJK-16S, eBioscience), F4/80 (clone: BM8, eBioscience), CD11b (clone: M1/70, eBioscience), Ly6G (clone: 1A8, eBioscience), CD206 (clone: 19.2, eBioscience), MHC-II (clone: M5/114.15.2, eBioscience), PD-L1 (clone: MIH5, eBioscience) and PD-1 (clone: RPM1-30, eBioscience) , Ly6C (clone: AL-21, BD Biosciences) and CCR2 (clone: 475301, R&D Systems) were used for FCM analysis. Mouse anti-PD-1 mAb (clone: J43, BioXCell) was used for PD-1 blocking in vivo. Mouse anti-TNFR1 mAb (clone: 55R-286, BD Pharmingen) was used for TNFR1 blocking in vitro. Mouse anti-CD3 (clone: 145-2C11, BioXCell) and anti-CD28 (clone: 37.51, BioXCell) antibodies were used for T stimulation in vitro. Mouse anti-CCL2 antibody (clone: ab25124, Abcam). Mouse antibody to CD11b (clone: ab133357, Abcam), CD31 (clone: ab28364, Abcam), F4/80 (clone: D2S9R, Abcam), CCL2 (clone: ab25124, Abcam) and TNFα (clone: ab6671, Abcam) were used for IHC staining.
Validation	All antibody used in the study were commercially available and validated by manufacturers.

Eukaryotic cell lines

Policy information about cell lines

Cell line source(s)	CT26, MC38 and Hepa1-6 cell lines sourced from ATCC.
Authentication	CT26 and MC38 was obtained from the Chinese Academy of Sciences, Shanghai Institutes for Biological Sciences (Shanghai, China). Hepa1-6 was obtained from Applied Biological Materials. Lnc (Zhenjiang, China).
Mycoplasma contamination	All cell lines tested negative for mycoplasma contamination by PCR.
Commonly misidentified lines (See ICLAC register)	No

Animals and other organisms

Policy information about studies involving animals; ARRIVE guidelines recommended for reporting animal research

Laboratory animals	8-12 weeks old male Balb/c (H2Kd) and C57BL/6 (B6; H2Kb) mice were used in this study.

Wild animals	The study did not involve wild animals.
Field-collected samples	Not applicable
Ethics oversight	All experimental protocols were approved by Committee for the Protection of Animal Care Committee at Soochow University.

Note that full information on the approval of the study protocol must also be provided in the manuscript.

Flow Cytometry

Plots

Confirm that:

- [x] The axis labels state the marker and fluorochrome used (e.g. CD4-FITC).
- [x] The axis scales are clearly visible. Include numbers along axes only for bottom left plot of group (a 'group' is an analysis of identical markers).
- [x] All plots are contour plots with outliers or pseudocolor plots.
- [x] A numerical value for number of cells or percentage (with statistics) is provided.

Methodology

Sample preparation	Tumor masses were removed, homogenized, and digested with collagenase and hyaluronidase solution. The resulting cell suspension was filtered through a cell mesh and resuspended in Hank's media plus 1% fetal calf serum (FCS) for further analysis.
Instrument	FCM analysis was performed using a FACS flow cytometer (Canto II, BD). T
Software	Data were analyzed using FlowJo software (Treestar).First a FSC/SSC-plot was made and we gated all leukocytes.
Cell population abundance	Cell suspension was adjusted to 5.0×106/mL by counting plate, and 200μL cell suspension was added into each EP tube.
Gating strategy	First a FSC/SSC-plot was made and we gated all leukocytes. The boundaries between "positive" and "negative" staining cell population were defined according negative control.

- [ ] Tick this box to confirm that a figure exemplifying the gating strategy is provided in the Supplementary Information.
